# Reverse Aqua Pump Negative Pressure Wound Therapy: A Noninferiority Randomized Controlled Trial of Effectiveness and Affordability in Optimizing the Inflammatory and Proliferative Phases of Wound Healing in Developing Countries

**DOI:** 10.1016/j.jhsg.2026.101032

**Published:** 2026-06-17

**Authors:** Meirizal Meirizal, Hilmi Muhammad, Agung Susilo Lo, I. Made Dolly

**Affiliations:** ∗ Orthopedics and Traumatology Division, Surgery Department, RSUP Dr. Sardjito Hospital, Universitas Gadjah Mada, D.I. Yogyakarta, Indonesia; † Faculty of Medicine, Public Health and Nursing, Universitas Gadjah Mada, D.I. Yogyakarta, Indonesia

**Keywords:** Cost-benefit analysis, Developing countries, Negative pressure wound treatment, Randomized controlled trial, Treatment outcome

## Abstract

**Purpose:**

Negative pressure wound treatment (NPWT) has been proven effective in accelerating wound healing. However, its high cost limits its use in developing countries, and innovations have largely focused on supplementary rather than core system components. The reverse aqua pump (RAP)-NPWT was developed as a low-cost alternative with effectiveness equivalent to commercial NPWT. This study aims to assess clinical outcomes and analyze the costs of using RAP-NPWT in patients requiring further reconstruction wounds with skin grafts.

**Methods:**

A noninferiority randomized controlled trial design comparing RAP-NPWT with commercial NPWT through comparative outcome, correlation analysis, and cost evaluation. A total of 24 subjects (12 RAP-NPWT and 12 NPWT control) were analyzed with no significant differences in baseline characteristics.

**Results:**

No significant difference was found in the degree of granulation from the first day to the end of treatment. Bone exposure did not affect granulation development, and the duration of NPWT use between groups did not differ. Economically, RAP-NPWT showed significantly lower treatment costs with a negative incremental cost-effectiveness ratio and cost-minimization analysis, placing it as the dominant intervention that is both more cost effective.

**Conclusions:**

Overall, RAP-NPWT provides noninferior clinical efficacy to commercial NPWT but at a significantly lower cost, making it worthy of consideration as a more affordable solution in developing countries.

**Type of study/level of evidence:**

Randomized controlled trial IB.

Wound management presents a considerable clinical challenge owing to the variety in wound features, patient comorbidities, and unpredictable healing patterns. Chronic wounds provide a significant global health and economic challenge, especially among elderly populations and those with chronic illnesses.^1^^,^^2^ The rising demand for sophisticated wound-care solutions illustrates the importance of treatments that are both clinically effective and economically viable.^3^

Negative pressure wound therapy (NPWT), first introduced by Argenta and Morykwas^4^ in 1997, has become a cornerstone in modern wound management. NPWT stimulates angiogenesis and granulation tissue formation, reduces edema, enhances perfusion, and removes exudate by applying controlled subatmospheric pressure. In comparison to conventional dressings, systematic evaluations have shown that wound healing outcomes are significantly improved, particularly in complex and traumatic wounds.^5^ NPWT has become a widely accepted procedure for wound-bed preparation prior to definitive reconstruction because of these physiological and clinical advantages (Figure 1, Figure 2, Figure 3).

**Figure 1:**
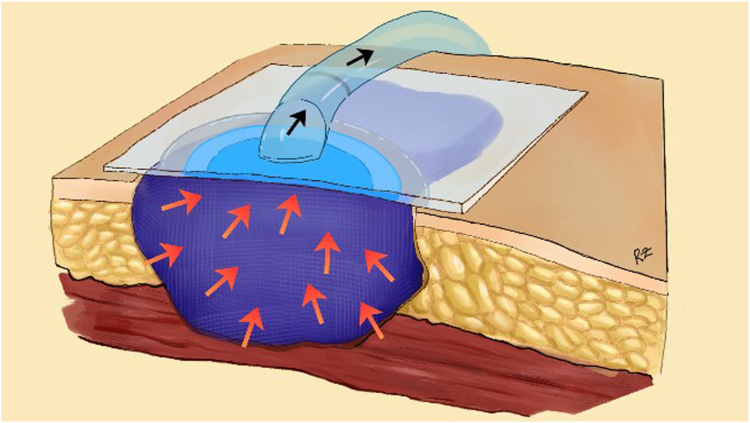
Aquarium pump mechanism in RAP-NPWT.

**Figure 2:**
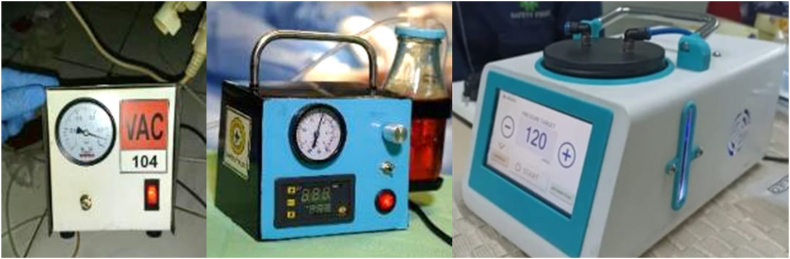
Mechanism of action in RAP-NPWT.

**Figure 3:**
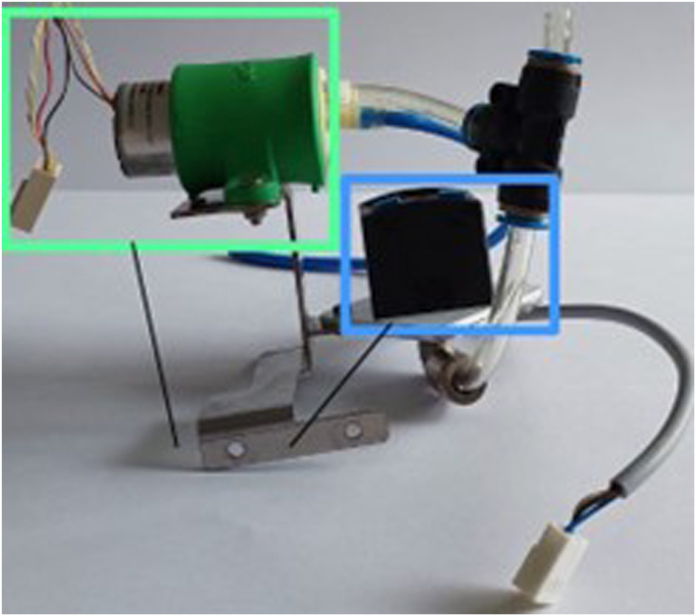
The development of RAP-NPWT over the years to the latest version.

However, the clinical benefits of NPWT are often offset by its high operational cost. Commercial systems require device rental fees and specialized disposable components, contributing to high per-episode treatment expenses.^6^ Low-cost NPWT systems in low- and middle-income countries have shown comparable wound healing outcomes while significantly reducing costs. However, most rely on modified components or hospital infrastructure rather than redesigning the core pressure mechanism, limiting accessibility.^7^^,^^8^ In our hospital, which is a tertiary-referral hospital, the cost of a single commercial NPWT unit remains prohibitive for many patients, excluding additional consumable materials. To address these limitations, several low-cost modifications have been proposed, such as wall suction-based systems^9^ or syringe-generated negative pressure.^10^ Although these alternatives aim to reduce financial burden, many focus primarily on modifying supplementary materials rather than redesigning the core pressure-generating mechanism. Moreover, high-quality randomized controlled trials evaluating both clinical noninferiority and formal cost effectiveness of such modified systems remain limited.^11^

To overcome such challenges, a novel Reverse Aqua Pump-NPWT (RAP-NPWT) system has been developed since 2019. By addressing both effectiveness and economic impact, this trial aims to provide evidence for a practical and affordable NPWT alternative suitable for resource-limited health care settings.

## Materials and Methods

### Study design

This study was designed as a noninferiority randomized controlled trial with a 1:1 allocation ratio, comparing the clinical and economic outcomes of RAP-NPWT with a commercial NPWT system. Computerized randomization was conducted at the individual patient level with concealed allocation and assessor blinding. The design followed the CONSORT 2025 recommendations for noninferiority randomized controlled trials.

### Trial setting

The trial was conducted from April to August 2025 at tertiary-referral hospital at Daerah Istimewa Yogyakarta, Indonesia. Recruitment took place in inpatient, outpatient, and surgical settings. This clinical trial was registered with the Indonesia Clinical Research Registry (INA-CRR). The details are as follows: Registry Number: INA-3631E32

### Sample size

The sample size was calculated using the noninferiority randomized controlled trial formula previously described.^12^ The noninferiority margin was defined as the cost difference between the RAP-NPWT and the commercial NPWT system. Based on a one-sided significance level (α) of 0.05 (Z = 1.645) and a power of 80% β = 0.20 (Z = 0.845), the required sample size was determined to be 12 participants per group, totaling 24 participants. Participants were recruited consecutively according to the inclusion and exclusion criteria.

### Participants

Eligible participants were adults aged 17–75 years presenting with soft-tissue defects requiring reconstructive procedures and planned for lower-limb skin grafting. Exclusion criteria included refusal to undergo debridement, uncontrolled diabetes or hypertension, peripheral vascular disease, malignancy, autoimmune disease, and wounds requiring flap reconstruction. All interventions were performed by the same surgical and wound-care team certified in NPWT.

### Intervention and comparator

The intervention group received wound care using the Reverse Aqua Pump-NPWT, an innovative low-cost device, whereas the control group received a standard commercial NPWT. Both systems applied negative pressure of –125 mm Hg continuously until exudate production was <100 mL/24 hours, followed by intermittent suction (5 minutes on, 2 minutes off) with the same pressure. Dressings were changed twice weekly and replaced every 4 days. The end point was the time required to achieve optimal granulation for secondary skin grafting. Both groups received identical wound-care regimens.

### Outcomes

The primary outcomes were wound granulation formation, duration to optimal granulation, and cost effectiveness. Economic end points were selected because the high cost of NPWT remains the principal barrier to its implementation in developing countries. Therefore, evaluating treatment cost, cost per treatment episode, and cost effectiveness was essential to determine the feasibility, affordability, and potential scalability of RAP-NPWT as a practical alternative in resource-limited health care settings. Wound granulation was defined as newly formed vascularized connective tissue consisting of capillaries, inflammatory cells, fibroblasts, and extracellular matrix, clinically appearing as reddish granular tissue covering the wound bed. The granulation area was measured in square centimeters using the validated IMITO mobile application following surgical debridement and during each dressing change until complete granulation was achieved and the wound was ready for skin grafting. Wound-care cost was calculated based on total expenditure for consumable medical materials used during NPWT therapy, including both RAP-NPWT and commercial NPWT systems. Harms and adverse events (pain, bleeding, necrosis, and new infection) were monitored systematically during dressing changes.

### Blinding

Outcome assessors were blinded to group allocation. Participants were aware of the type of device used, but both NPWT systems had similar dressing appearance to minimize bias. Participants were enrolled by the surgical team, whereas intervention assignment was performed by an independent research assistant. Data analysts were blinded during statistical analysis.

### Data collection

Baseline demographic and clinical data were recorded in standardized case report forms. Wound dimensions and granulation were measured using the validated IMITO mobile application.^13^ Inflammatory markers were assessed at baseline and postintervention days 4 and 6. Cost data included device use and consumable materials.

### Statistical analysis

Analyses normality was tested with the Kolmogorov–Smirnov test. Normally distributed data were compared using independent *t* tests, and nonnormally distributed data were compared using Mann–Whitney tests. Subgroup analyses were performed by wound base type. Cost effectiveness was evaluated using the incremental cost-effectiveness ratio (ICER) and cost-minimization analysis. A *P* value < .05 was considered statistically significant.

### Protocol changes and ethics

The study was approved by Institution Ethics Committee of the institution where the research was conducted (KE/FK/0638/EC). Written informed consent was obtained from all participants. The RAP-NPWT device was registered with the Indonesian Medical Device Authority (BPAFK). No major adverse events occurred during the trial (KE/FK/0638/EC).

## Results

### Subject characteristics

**Figure 4:**
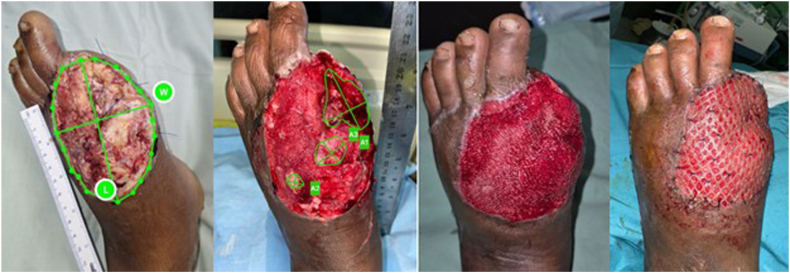
A 31-year-old man with a left foot wound defect treated with NPWT control for 15 days.

**Figure 5:**
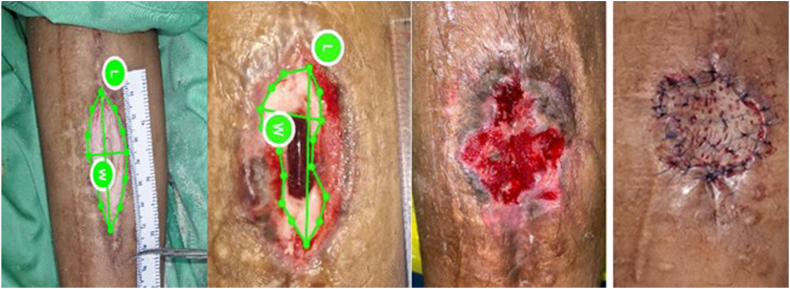
An 18-year-old man with a right cruris wound defect treated with RAP-NPWT for 36 days.

**Figure 6:**
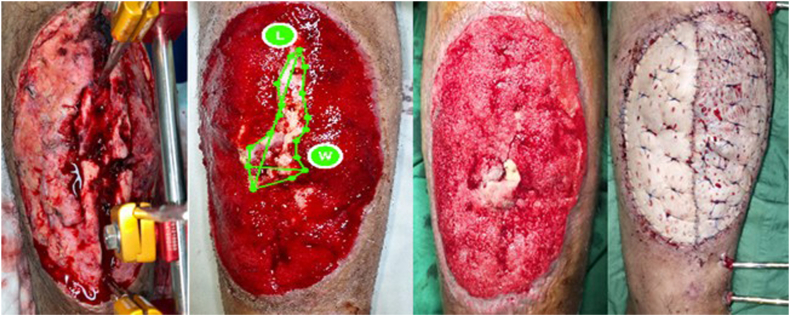
An 26-year-old man with a left cruris wound defect treated with RAP-NPWT for 60 days.

A total of 24 subjects were included and equally divided into the RAP-NPWT group and NPWT control group. All wounds were located in the lower extremities, predominantly involving the foot and cruris regions. Despite slight differences in wound base, the overall demographic and clinical profiles remained comparable (Table 1). These findings indicate that both groups started with relatively equal clinical conditions, allowing for a fair comparison of outcomes. Documentations of a few subjects of wound progression in the RAP-NPWT and NPWT control groups were conducted until both groups achieved complete granulation and underwent skin grafting (Figure 4, Figure 5, Figure 6).

**Table 1:** Subject Characteristics

Variable	RAP-NPWT N = 12	NPWT Control N = 12	Statistical Analysis	*P* Value
Sex				
M	7	8	Chi square	.571
F	5	4		
Wound Base				
Muscle/fascia	5	11	Chi square	.025
Bone exposed	7	1		
Age (y)	35.25 ± 20.12	41.46 ± 14.23	Shapiro–Wilk	.067
Albumin (g/dL)	3.74 ± 0.52	3.61 ± 0.43	Shapiro–Wilk	.165
Wound H0 (cm^2^)	49.01 ± 31.57	41.21 ± 28.42	Shapiro–Wilk	.031
Wound HE (cm^2^)	42.39 ± 31.68	39.25 ± 27.37	Shapiro–Wilk	.012
Duration (d)	19.66 ± 9.56	19.69 ± 8.40	Shapiro–Wilk	.071

### Wound granulation

Granulation outcomes were analyzed across sequential dressing sessions up to the tenth dressing, which represented the last common observation point before most patients proceeded to definitive reconstruction. The comparison of wound granulation formation showed no statistically significant difference at any time point (Table 2). During the early dressing periods (dressing one to dressing four), the mean wound granulation formation was faster. Both groups showed a relatively higher average granulation area in this period. The granulation area then began to decline in both groups, indicating that the granulation process began to plateau as the wound approached readiness for the next phase of healing. This indicates that the pattern of initial granulation acceleration and final stage decline appeared similar in between two groups.

**Table 2:** Wound Granulation Formation

Dressing	N	RAP- NPWT	NPWT Control	Mann–Whitney	*P* Value
		Mean (cm^2^)			
Dressing 1	24	36.07	27.42	71.0	.703
Dressing 2	24	40.49	30.72	66.0	.750
Dressing 3	23	36.89	42.35	53.0	.457
Dressing 4	19	40.02	29.23	41.0	.744
Dressing 5	12	43.21	35.50	17.0	.873
Dressing 6	8	20.98	31.90	6.0	.564
Dressing 7	6	17.91	28.47	4.0	.827
Dressing 8	2	7.83	20.38	0.0	.317
Dressing 9	2	7.90	20.35	0.0	.317
Dressing 10	2	9.98	20.36	0.0	.317

The subgroup analysis was performed after obtaining an overall granulation rate of 96.61%, and the results showed variations in granulation. The principal coefficient for the granulation rate reached 96.61 with significance (*P* < .001), indicating that the granulation process generally proceeded well in all samples. Meanwhile, the use of RAP-NPWT did not show a significant effect on the extent of granulation. The wound with exposed bone did tend to decrease granulation but was not statistically significant (Table 3). These findings confirm that both interventions provide comparable results and high percentage of granulation formation.

**Table 3:** Subgroup Wound Granulation Analysis

Variable	B (Coefficient)	Standard Error	*P* Value
Granulation (%)	96.61	2.68	<.001
RAP-NPWT	2.24	4.19	.954
Bone exposed	–5.46	4.36	.223

### Wound treatment evaluation

Evaluation between the RAP-NPWT and NPWT groups showed that wound size, duration, and noise level were not significantly different. However, a difference was observed with RAP-NPWT, showing significantly lower total cost of treatment than conventional NPWT (Table 4). These findings confirm that despite the similar clinical effectiveness of both methods, RAP-NPWT offers substantial economic advantages, making it a more affordable alternative for wound management.

**Table 4:** Wound Treatment Evaluation

Variable	Statistical Analysis	RAP- NPWT	NPWT Control	*P* Value
		Mean ± SD		
Wound Size (cm^2^)	Mann–Whitney	49.01 ± 31.57	41.21 ± 28.42	.514
Duration (d)	*t* Test	35.25 ± 20.12	41.46 ± 14.23	.805
Total Cost (IDR)	Mann–Whitney	1,590,541.66 ± 773,678.97	6,648,615.38 ± 2,836,132.44	**<.001**
Decibel (dB)	*t* Test	48.83 ± 6.36	49.53 ± 4.78	.756

Bold values indicate statistical significance (*P* < .05).

### Cost evaluation

Correlation analysis results appear to show that the relationship between wound size and treatment duration and costs did not show a statistically significant correlation in either the RAP-NPWT group or the NPWT control group (Table 5). These findings indicate costs were not influenced by wound size or duration of NPWT.

**Table 5:** Correlation Wound Size to Duration and Total Cost

Variable		Statistical Analysis	RAP-NPWT	NPWT Control
			Correlation Coefficient	
Wound size	Duration	Spearman correlation	–0.342 *P* = .276	–0.284 *P* = .346
Wound size	Cost	Spearman correlation	–0.389 *P* = .211	–0.273 *P* = .368

### ICER

The use of RAP-NPWT was significantly more economical than commercial NPWT, with an average expenditure of approximately USD 94.25 ± 45.84 compared to USD 393.97 ± 168.06. In terms of effectiveness, RAP-NPWT also provided a greater reduction in wound size (6.62 cm^2^) than commercial NPWT (1.96 cm^2^), and the duration of device use was relatively the same at 19.66 ± 9.56 days compared to 19.69 ± 8.40 days. These findings confirm that RAP-NPWT represents a more cost-effective alternative to commercial NPWT.

ΔCost=CRAP-NPWT–CNPWTControl

=USD94.25−USD393.97

=USD−299.72

ΔEffectiveness=ERAP-NPWT–EControl

$$=6.62\;–\;1.96\;{\text{cm}}^{2}$$

$$=4.66\;{\text{cm}}^{2}$$

ICER=ΔCost/ΔEffectiveness

$$=\text{USD}-299.72/\;4.66\;{\text{cm}}^{2}$$

$$=\text{USD}-64.32\;\text{every}\;1\;{\text{cm}}^{2}\;\text{reduces}\;\text{wound}\;\text{size.}$$

### Cost-minimization analysis

The clinical outcomes between RAP-NPWT and commercial NPWT were statistically equivalent in wound size and granulation formation. A cost-minimization approach was applied to quantify the financial advantage assuming equal therapeutic benefit. To illustrate efficiency, the cost per day of therapy was also computed:

Cost/dayRAP-NPWT=USD94.25/19.66=USD4.79/day

Cost/dayNPWTControl=USD393.97/19.69=USD20.01/day

RAP-NPWT therefore reduces daily wound management expenditure by approximately USD 15.22/day or USD −299.72 per treatment episode, representing a 4.2-fold economic improvement from the provider’s standpoint.

## Discussion

This study shows RAP-NPWT and commercial NPWT provide equivalent clinical outcomes in terms of wound granulation growths, characterized by acceleration in the early phase and plateau in the late phase, with no significant differences at any assessment point. Although the clinical outcomes are not different, RAP-NPWT provides an economic advantage with cost reduction.

Reverse aqua pump-negative pressure wound treatment previously reported was applied to a mangled injury with a Mangled Extremity Severity Score (MESS) of nine and successfully promoted rapid granulation formation in the exposed critical structures, allowing definitive closure with a skin graft and limb salvage at 1 year.^14^ A case series of 13 patients with various soft-tissue defects (4.28–111.2 cm^2^) achieved complete granulation by the end of therapy with a relatively homogeneous wound-bed preparation time of 18 to 26 days.^15^

A study of early RAP-NPWT in 2019 demonstrated granulation time ranged from 7 to 60 days, with most cases achieving optimal granulation within 10–30 days, although some severe wounds required longer durations.^16^ The duration of NPWT therapy in this study, approximately 35–41 days, was generally slightly longer than reported in the previous literature, likely reflecting the complexity of wound size variation and the more standardized dressing protocols in the randomized controlled trial. All these findings indicate that RAP-NPWT produces stable and effective wound granulation growth with different wound severities and different study designs.

The granulation pattern in this study demonstrated an early acceleration then plateau; this aligns with the physiological phases of wound healing. During the early proliferative phase, fibroblast proliferation, angiogenesis, and extracellular matrix deposition drive rapid granulation tissue formation, resulting in increased initial granulation rate.^17^^,^^18^ As the wound bed becomes progressively filled and approaches readiness for skin grafting, granulation activity naturally slows, forming a plateau phase.^18^ RAP-NPWT and the commercial NPWT exhibited expected granulation trajectories further supporting the conclusion that the two systems provide equivalent clinical performance in facilitating wound-bed preparation.^18^^,^^19^

Wound granulation is a critical step in preparing soft-tissue defects for definitive coverage, and NPWT has been recognized as an effective method to optimize this phase. NPWT enhances perfusion, reduces edema, and stimulates micro- and macrodeformation that induce granulation. Continuous removal of exudate further promotes a favorable biological environment for tissue growth.^19^, ^20^, ^21^ These well-established physiological effects provide the foundation for evaluating alternative NPWT systems, including modified or low-cost designs employing similar pressure dynamics.

Previous studies have shown various NPWT systems, including commercial and non-commercial systems as well as wall suction-based systems, produced no significant differences in granulation formation or wound size reduction, indicating that effectiveness is more influenced by the application protocol than device branding.^22^, ^23^, ^24^

The cost of commercial NPWT remains a major barrier in low- to middle-income countries, which would benefit from lower-cost dressing modifications and simplified systems.^25^^,^^26^ In this cost analysis, the correlation among treatment costs, wound size, and duration of therapy did not show statistical significance, and the coefficient showed a weak and negative relationship, suggesting that the lower costs in the RAP-NPWT group were not simply an effect of smaller wounds or shorter duration of therapy. These findings support that the use of low-cost versions of NPWT such as RAP-NPWT may offer intrinsic cost savings without compromising clinical effectiveness.

The ICER calculation shows that RAP-NPWT is a dominant intervention both clinically and economically compared to commercial NPWT. Cost-minimization analysis shows daily therapy costs of USD 4.79 for RAP-NPWT compared with USD 20.01 for commercial NPWT, representing a 4.2-fold efficiency and potential savings of USD 2,992 per month for every 10 patients. Therefore, RAP-NPWT, as a modification, can provide economic benefits without compromising clinical outcomes, consistent with the previous literature.^6^^,^^27^

Typical sound levels, particularly in intensive care unit and emergency departments, often remained between 45 and 60 dB.^28^^,^^29^ Earlier study has also emphasized that elevated noise from medical equipment can contribute to patient discomfort and increased stress among health care staff, underscoring the importance of maintaining acceptable device noise levels.^30^ The similar noise profile between RAP-NPWT and commercial NPWT supports the interpretation that the system maintains acceptable acoustic performance while still offering other advantages demonstrated in this study.

Several limitations should be acknowledged. This economic evaluation exclusively adopted the provider perspective, excluding indirect costs such as transportation, productivity loss, or caregiver burden. Consequently, the broader cost effectiveness of RAP-NPWT remains to be determined. Future multicenter studies with larger cohorts, longer follow-up, and inclusion of quality-adjusted life years or patient-reported outcomes are planned to confirm these results.

## Ethical Approval

This research has received approval from the Ethics Committee of the Faculty of Medicine, Universitas Gadjah Mada, D. I. Yogyakarta, with ethical clearance approval letter number KE/FK/0638/EC/2025.

## Consent for Publication

Written informed consent was obtained from all participants prior enrollment in this randomized controlled trial. All participants were informed about the study objectives, procedures, potential risks and right to withdraw at any time without penalty. Documentation of the informed consent process is available for review by the Editor-in-Chief of this journal upon request.

## Conflicts of Interest

No benefits in any form have been received or will be received related directly to this article.
